# The Causal Role of Mitochondrial Dynamics in Regulating Innate Immunity in Diabetes

**DOI:** 10.3389/fendo.2020.00445

**Published:** 2020-07-29

**Authors:** Yen-Hsiang Chang, Hung-Yu Lin, Feng-Chih Shen, Yu-Jih Su, Jiin-Haur Chuang, Tsu-Kung Lin, Chia-Wei Liou, Ching-Yi Lin, Shao-Wen Weng, Pei-Wen Wang

**Affiliations:** ^1^Department of Nuclear Medicine, Kaohsiung Chang Gung Memorial Hospital and Chang Gung University College of Medicine, Kaohsiung, Taiwan; ^2^Center for Mitochondrial Research and Medicine, Kaohsiung Chang Gung Memorial Hospital, Kaohsiung, Taiwan; ^3^Department of Internal Medicine, Kaohsiung Chang Gung Memorial Hospital and Chang Gung University College of Medicine, Kaohsiung, Taiwan; ^4^Department of Surgery, Kaohsiung Chang Gung Memorial Hospital and Chang Gung University College of Medicine, Kaohsiung, Taiwan; ^5^Department of Neurology, Kaohsiung Chang Gung Memorial Hospital and Chang Gung University College of Medicine, Kaohsiung, Taiwan

**Keywords:** mitochondrial dynamics, innate immunity, type 2 diabetes, nutrient excess, inflammation

## Abstract

**Background:** Plenty of evidence suggested that chronic low-grade inflammation triggered by innate immunity activation contributes to the pathogenesis of type 2 diabetes (T2D). Using the trans-mitochondrial cybrid cell model, we have demonstrated that mitochondria independently take part in the pathological process of insulin resistance (IR) and pro-inflammatory phenotype in cybrid cells harboring mitochondrial haplogroup B4, which are more likely to develop T2D. The mitochondrial network is more fragmented, and the expression of fusion-related proteins is low in Cybrid B4. We also discovered the causal role of mitochondrial dynamics (mtDYN) proteins in regulating IR in this cybrid model, and the bidirectional interaction between mtDYN and mitochondrial oxidative stress is considered etiologically important. In this study, we further investigated whether mtDYN bridges the gap between nutrient excess and chronic inflammation in T2D.

**Methods:** Trans-mitochondrial cybrid cells derived from the 143B human osteosarcoma cell line were cultured in a medium containing glucose (25 mM) with or without saturated fatty acid (0.25 mM BSA-conjugated palmitate), and the expression of innate immunity/inflammasome molecules was compared between cybrid B4 (the major T2D-susceptible haplogroup among the Chinese population) and cybrid D4 (the major T2D-resistant haplogroup among the Chinese population). We investigated the causal relationship between mtDYN and nutrient excess-induced inflammation in cybrid B4 by genetic manipulation of mtDYN and by pharmacologically inhibiting mitochondrial fission using the Drp1 inhibitor, mdivi-1, and metformin.

**Results:** Under nutrient excess with high fatty acid, cybrid B4 presented increased mitochondrial pro-fission profiles and enhanced chronic inflammation markers (RIG-I, MDA5, MAVS) and inflammasome (NLRP3, Caspase-1, IL-1β), whereas the levels in cybrid D4 were not or less significantly altered. In cybrid B4 under nutrient excess, overexpression of fusion proteins (Mfn1 or Mfn2) significantly repressed the expression of innate immunity/inflammasome-related molecules, while knockdown had a less significant effect. On the contrary, knockdown of fission proteins (Drp1 or Fis1) significantly repressed the expression of innate immunity/inflammasome-related molecules, while overexpression had a less significant effect. In addition, Drp1 inhibitor mdivi-1 and metformin inhibited mitochondrial fission and attenuated the pro-inflammation expression as well.

**Conclusion:** Our results discovered the causal relationship between mtDYN and nutrient excess-induced chronic inflammation in a diabetes-susceptible cell model. Targeting mtDYN by direct interfering pro-fission can be a therapeutic intervention for chronic inflammation in T2D.

## Introduction

Type 2 diabetes (T2D), clustering with a group of risk factors of metabolic origin, increases cardiovascular disease risk (1). Recently, it has been highlighted that mitochondrial dysfunction, oxidative stress, and chronic inflammation serve as upstream events leading to diabetes and its complications in affected tissues (2–6). Plenty of evidence suggested that chronic low-grade inflammation triggered by innate immunity activation contributes to the pathogenesis of T2D (7–9). A variety of pathogen- and host-derived “danger” signal may activate the nucleotide oligomerization domain (NOD)-like receptor family pyrin domain containing 3 (NLRP3) inflammasome (10, 11). The well-known endogenous stress and injury-related products include adenosine triphosphate (ATP) (injury and necrotic cell death), amyloid-β fibril (Alzheimer's disease), high glucose levels (metabolic syndrome), and monosodium urate (gout) (10, 11).

Mitochondrial dysfunction has been regarded as a fundamental factor in the triggering of NLRP3-mediated inflammation (12), and mitochondrial reactive oxygen species (ROS) overproduction is critical for activation of NLRP3 inflammasome (13). Mitochondrial dynamics (mtDYN), which refers to repeated cycles of mitochondrial fusion and fission, is a quality-control system that serves to optimize mitochondrial function (14). Studies have demonstrated the involvement of mtDYN in cellular energy homeostasis (15, 16), cell cycle (17), apoptosis (18–20), oxidative phosphorylation (21), oxygen consumption (22), ROS (23), and autophagy (24, 25). In our previous studies using the trans-mitochondrial cybrid cell model, we have demonstrated that mitochondria independently take part in the pathological process of insulin resistance (IR) and pro-inflammatory phenotype in cybrid cells harboring mitochondrial haplogroup B4, which are more likely to develop T2D in the Chinese population (3, 26). The mitochondrial network is more fragmented, and the expression of fusion-related proteins is low in Cybrid B4 (4). By using this cybrid model, we also found the causal role of mtDYN in the development of IR from T2D-susceptible mitochondrial haplogroup, and the bidirectional interaction between mtDYN and mitochondrial oxidative stress is considered etiologically important (5). However, the relationship between mtDYN and innate immunity/inflammasome in T2D remains unknown. In this study, we further investigated whether mtDYN bridges the gap between nutrient excess and chronic inflammation in T2D.

## Methods

### Cybrid Cell Generation

Our culture system has been described previously (27). Firstly, mtDNA-depleted ρ^0^ cells were generated by exposing 143B osteosarcoma cells (ATCC® CRL-8303™, purchased from Bioresource Collection and Research Center (BCRC), Hsinchu, Taiwan) to low-dose ethidium bromide, followed by single-clone isolation. Next, trans-mitochondrial cybrid cells were generated by fusing 143B-ρ^0^ cells with human platelets which were isolated from volunteer subjects harboring mtDNA haplogroup B4 or D4. Fusion of platelets with ρ^0^ cells was conducted in the presence of polyethylene glycol 1500 (50% w/v; Roche, Nutley, NJ, USA). Cybrid cells were cultured in Dulbecco's Modified Eagle Medium (DMEM; 11995-065, Gibco) containing 10% fetal bovine serum (FBS; 04-001-1A, Biological Industry), in an incubator with 5% CO_2_ at 37°C. The studies involving human participants were reviewed and approved by the Institutional Review Board of Chang Gung Memorial Hospital (CGMH; IRB number 101-1620A3). Written informed consent was obtained from the participants of the study.

### Overexpression of Mfn-1, Mfn-2, Fis-1, and Drp-1, siRNA Knockdown and Treatment of Drugs

Gene overexpression of Mfn1, Mfn2, Drp1, and Fis1 (RG207184; RG202218; RG202046; RG202560; Origene Technologies, Inc., Rockville, MD, USA) were performed using Lipofectamine® 2000 (1367620, Invitrogen) in the presence of Opti-MEM (31985-070, Gibco) for 18 h. A DNA (μg) to Lipofectamine® 2000 (μl) ratio of 1:1 was used for transfection. GFP-expression vector (OriGene-ORIPS100010, Origene) was used as mock control (Technologies, Inc., Rockville, MD, USA). For siRNA knockdown, interfering RNAs (sc-43927; sc-43928; sc-43732; sc-60643; Santa Cruz Biotechnology, Santa Cruz, CA) were delivered using Lipofectamine® 2000 (1367620, Invitrogen) in the presence of Opti-MEM (31985-070, Gibco) for 4 h, followed by washing and replacement with a fresh growth medium for 72 h. We grew cybrid cells individually under 25 mM glucose or glucose plus saturated fatty acid (FA) for 6 h to create different nutritional conditions. Saturated fatty acid palmitate (P9767; Sigma) was conjugated into bovine serum albumin as a carrier. The concentration of palmitate was 0.25 mM, similar to postprandial levels in humans (28). For drug treatment experiments, cells were treated with vehicle, 1 mM metformin (M0605000, Sigma-Aldrich), or 10 μM mdivi-1 (M0199, Sigma-Aldrich) for another 24 h.

### Western Blotting

The cells were seeded at a density of 5 × 10^6^ cells per 100-mm dish (Nunc, Denmark). The cells were harvested, after which their protein extract was isolated using a buffer containing 150 mM NaCl, 50 mM HEPES pH 7, 1% Triton X-100, 10% glycerol, 1.5 mM MgCl_2_, 1 mM EGTA, and a protease inhibitor. Following centrifugation, the concentration of the protein lysate was measured by Bradford Protein Assay Kit (#23200, Thermo Fisher). The protein for SDS-PAGE was then prepared with 2× solution (4% SDS, 20% glycerol, 10% 2-mercaptoethanol, 0.004% bromophenol blue, and 0.125 M Tris–HCl) and heated in boiling water for 5 min. 20 μg proteins was loaded and separated via SDS-PAGE by using an 8–10% polyacrylamide gel in an electrophoresis chamber (Mini-PROTEAN® Tetra Vertical Electrophoresis Cell, Bio-Rad), and then they were transferred onto a polyvinylidene fluoride (PVDF) membrane (Millipore) by using a transfer apparatus (Power Blotter–Semi-dry Transfer System, Thermo Fisher). The protein-loaded PVDF membrane was blocked with 5% skimmed milk in TBS-T for 1 h at room temperature and then incubated overnight at 4°C with primary antibodies including anti-RIG-1 (PA5-20276, Thermo, 1:1000), anti-NLRP3 (15101S, Cell Signaling, 1:1000), anti-caspase-1 (3866S, Cell signaling, 1:1000), anti-MDA5 (PA5-20337, Thermo Fisher, 1:1000), anti-MAVS (PA5-20348, Thermo Fisher, 1:1000), anti-MFN2 (ABC42, Millipore, 1:1000), anti-MFN1 (ABC41, Millipore, 1:1000), anti-p-DRP1 (3455S, Cell Signaling, 1:1000), anti-FIS1 (sc-376447, Santa Cruz, 1:1000), and anti-β-actin (sc-47778, Santa Cruz, 1:1000). Further, following conjugation of the secondary antibodies with HRP including goat-anti rabbit (sc-2004, Santa Cruz, 1:5000) and anti-mouse IgG (A9044, Sigma-Aldrich, 1:10000) at room temperature for 60 min, the immunoreactivity on the membrane was detected by ECL Plus luminal solution (Advansta, USA), under a photodocumentation system (UVP BioSpectrum 810, Thermo Fisher).

### Measurement of Mitochondrial Function

Cellular metabolic liability assessed by oxygen consumption rate (OCR) and extracellular acidification rate (ECAR) was determined using a Seahorse XF24 Extracellular Flux Analyzer (Seahorse Bioscience Inc., Chicopee, MA, USA), as previously described (4). Briefly, 3 × 10^6^ cells per well were seeded in Seahorse Cell Culture 24-well plates. Cells were incubated in DMEM in Seahorse cell plates for 1 h before measurement. When assay was performed, four measurements of the basal level of oxygen consumption were recorded. Subsequently, oligomycin (0.75 μM), a complex V inhibitor, was injected and mixed, and three measurements were recorded to determine ATP-linked oxygen consumption. Following oligomycin, FCCP (0.2 μM), a proton uncoupler, was injected to determine maximal respiration capacity with another three measurements recorded. Finally, complex I inhibitor rotenone (0.8 μM) was injected for three measurements of non-mitochondrial oxygen consumption.

### Statistical Analysis

Data collected from at least three independent experiments were expressed as the mean ± standard error (*SE*). Statistical significance between groups was evaluated by one-way analysis of variance (ANOVA) followed by *post-hoc* Bonferroni's test. A *p-*value under 0.05 was considered as statistical significance. The statistic from experimental results was calculated using Microsoft Excel, and the graphs were plotted using GraphPad Prism software.

## Results

### Comparison of Mitochondrial Dynamics, Chronic Inflammation Markers, and Inflammasome in Cybrid B4 and D4 Cells Under Nutrition Excess and Metformin Treatment

**Figure 1:**
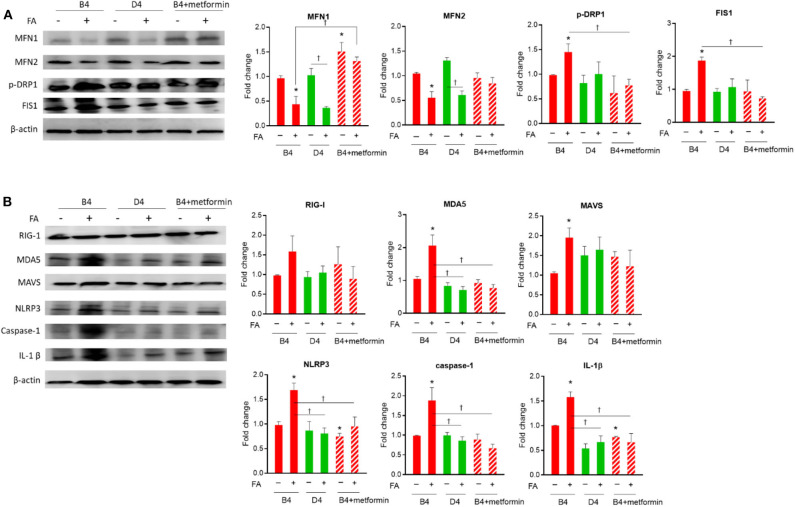
Expression profile of mitochondrial dynamic and innate immune markers in cybrid B4, D4, and B4 treated with metformin under nutrient excess. Cybrid cells B4, D4, and B4+metformin treated with or without 0.25 mM FA for 6 h, followed by 1 mM metformin treatment for a further 24 h. Representative blot image of mitochondrial dynamic **(A)** and innate immune markers **(B)** and corresponding densitometric results. β-Actin as loading control. *p < 0.05 compared to the B4 group in the absence of FA. ^†^*p* < 0.05 comparison between indicated groups. FA, fatty acid.

As shown in Figure 1A, under nutrient excess with glucose and high fatty acid, the levels of dynamic fusion proteins (Mfn1/Mfn2) were found to be lower in both diabetes-susceptible cybrid B4 and diabetes-resistant D4 cells. In contrast, the expression of fission proteins (Drp1 and Fis1) was significantly increased in cybrid B4, whereas the levels in D4 cells were not or less significantly altered. After treatment with antidiabetic agent metformin in B4, the nutrition excess-induced pro-fission was rescued by increased Mfn1 and decreased Drp1/Fis1 expression. As to innate immunity-related chronic inflammation markers (Figure 1B), within group comparison, MDA5, MAVS, NLRP3, Caspase-1, and IL-1β were found to be higher in B4 under high fatty acid treatment, whereas these markers in D4 cells were not altered. For the comparison between B4 and D4, upon high fatty acid treatment, the expression of MDA5, NLRP3, Caspase-1, and IL-1β was significantly increased in B4. After treatment with metformin in diabetes-susceptible B4 cells, the expression of nutrition excess-induced innate immunity/inflammasome molecules was ameliorated, including MDA5, NLRP3, caspase-1, and IL-1β.

### Mfn1 and Mfn2 Ameliorate Nutrient Excess-Induced Expressions of Innate Immunity/Inflammasome Molecules in Diabetes-Susceptible Cybrid B4

**Figure 2:**
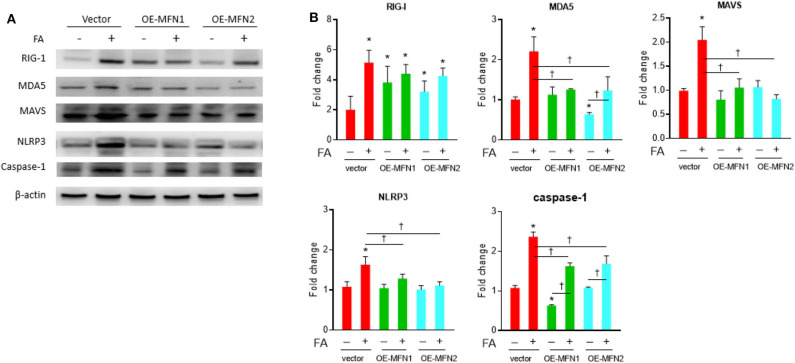
Role of MFN1/MFN2 overexpression on innate immune response in cybrid B4 under nutrient excess. Cybrid B4 cells were overexpressed with GFP, GFP-MFN1, or GFP-MFN2 for 18 h, followed by a 6-h exposure with or without 0.25 mM FA. **(A)** Representative blot image of RIG-I, MDA5, MAVS, NLRP3, and caspase-1. β-Actin as loading control. **(B)** Histograms of densitometric results. Quantification value of β-actin was used for normalization. *p < 0.05 compared to vector control in the absence of FA. ^†^*p* < 0.05 comparison between indicated groups. FA, fatty acid.

**Figure 3:**
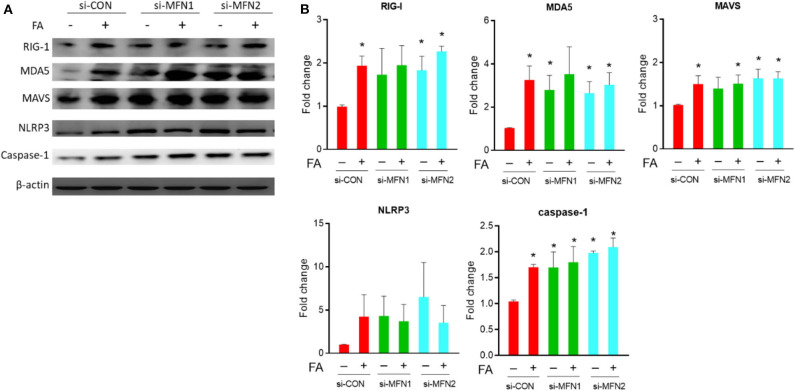
Role of MFN1/MFN2 knockdown on innate immune response in cybrid B4 under nutrient excess. **(A)** Representative blot image of RIG-I, MDA5, MAVS, NLRP3, and caspase-1. β-Actin as loading control. **(B)** Histograms of densitometric results. Quantification value of β-actin was used for normalization. *p < 0.05 compared to siRNA control in the absence of FA or between indicated groups. ^†^*p* < 0.05 comparison between indicated groups. CON, control; FA, fatty acid.

After overexpression of fusion-related proteins Mfn1 or Mfn2, the level of nutrient excess-induced innate immunity-related chronic inflammation markers, including MDA5, MAVS, NLRP3, and Caspase-1, was significantly reduced (Figure 2). This trend was observed in the presence of palmitate, while the trend was less significantly altered in the absence of palmitate. There was a significantly increased level of RIG-1, MDA5, MAVS, and Caspase-1 under basal nutrient conditions after knockdown of fusion-related molecules (Mfn1/Mfn2) (Figure 3). Overexpression significantly increased Mfn1 and Mfn2 protein abundance, whereas Mfn1 and Mfn2 were significant reduced after siRNA knockdown (Figure S1).

### Drp1 and Fis1 Deteriorate Nutrient Excess-Induced Expressions of Innate Immunity/Inflammasome Molecules in Diabetes-Susceptible Cybrid B4

**Figure 4:**
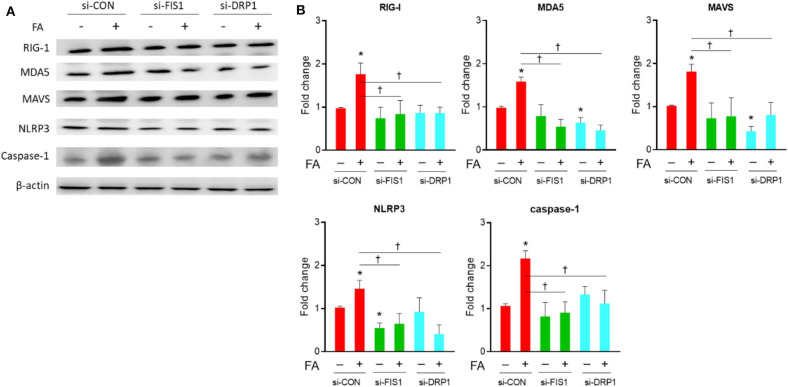
Role of DRP1/FIS1 knockdown on innate immune response in cybrid B4 under nutrient excess. **(A)** Representative blot image of RIG-I, MDA5, MAVS, NLRP3, and caspase-1. β-Actin as loading control. **(B)** Histograms of densitometric results. The quantification value of β-actin was used for normalization. *p < 0.05 compared to siRNA control in the absence of FA. ^†^*p* < 0.05 or between indicated groups. CON, control; FA, fatty acid.

**Figure 5:**
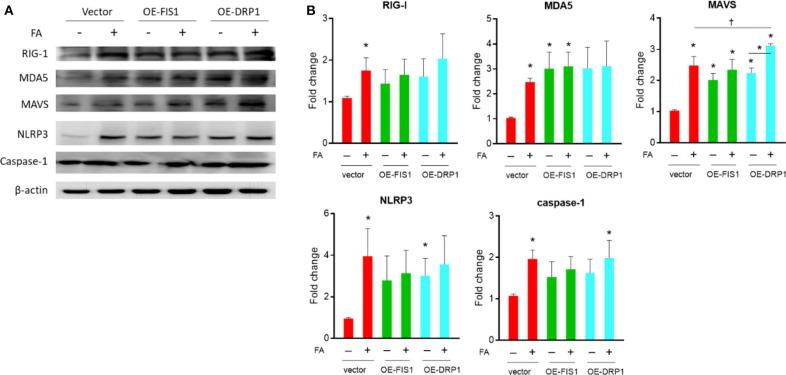
Role of DRP1/FIS1 overexpression on innate immune response in cybrid B4 under nutrient excess. **(A)** Representative blot image of RIG-I, MDA5, MAVS, NLRP3, and caspase-1. β-Actin as loading control. **(B)** Histograms of densitometric results. The quantification value of β-actin was used for normalization. *p < 0.05 compared to vector control in the absence of FA. ^†^*p* < 0.05 comparison between indicated groups. FA, fatty acid.

As shown in Figure 4, knockdown of fission-related proteins (Drp1/Fis1) significantly decreased the level of nutrient excess-induced innate immunity-related chronic inflammation markers, including RIG1, MDA5, MAVS, NLRP3, and Caspase-1. This trend was observed in the presence of palmitate, while the trend was less significant in the absence of palmitate. After overexpression of fission-related proteins (Drp1/Fis1), there was an increased MAVS level in the presence of absence of palmitate, while there was an increased MDA5 and NLRP3 level only under basal nutrient conditions (Figure 5). Overexpression significantly increased Drp1 and Fis1 protein abundance, whereas Drp1 and Fis1 were significant reduced after siRNA knockdown (Figure S1).

### Drp1 Inhibitor mdivi-1 Attenuated the Expression of Nutrient Excess-Induced Innate Immunity-Inflammasome in Diabetes-Susceptible Cybrid B4

**Figure 6:**
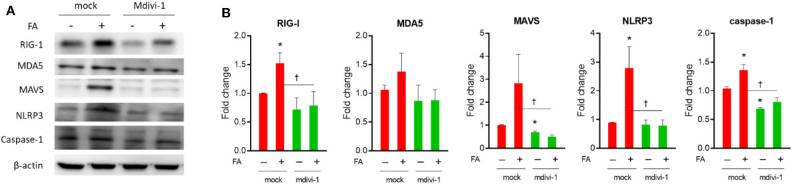
Effect of mdivi-1 on innate immune response in cybrid B4 under nutrient excess. Cybrid B4 cells were treated with/without FA for 6 h, followed by 10 μM mdivi-1 for another 24 h. **(A)** Representative blot image of RIG-I, MDA5, MAVS, NLRP3, and caspase-1. β-Actin as loading control. **(B)** Histograms of densitometric results. The quantification value of β-actin was used for normalization. *p < 0.05 compared to mock control in the absence of FA. ^†^*p* < 0.05 comparison between indicated groups. FA, fatty acid.

To validate the effect of pharmacological manipulation of mtDYN on the expression of a nutrient excess-induced innate immunity/inflammasome molecule, Drp-1 inhibitor, mdivi-1 was employed. The levels of RIG-1, MAVS, NLRP3, and caspase-1 were all significantly downregulated after mdivi-1 treatment (Figure 6).

### Perturbation of Mitochondrial Dynamics Affects Mitochondrial Energetics Under Different Nutrient Conditions

In the basal and maximal respiration panel, either Drp1 knockdown or Mdivi-1 can increase OCR under basal nutrient conditions, whereas the OCR was substantially lower after Mfn-1 knockdown. However, under nutrient excess conditions, Mfn-1 knockdown increased OCR, whereas the OCR was substantially lower after Drp1 knockdown and Mdivi-1 treatment than Mfn-1 knockdown (Figure S2).

## Discussion

The mitochondrion, the cellular powerhouse in all the eukaryotic organelle, plays a pivotal role in cellular metabolism (29), and mitochondrial dynamics (mtDYN) serves as a quality-control system essential for maintaining mitochondrial homeostasis (30). The dynamic behavior has now been reported to be associated with biosynthetic and bioenergetics characteristics (31). Plenty of evidence revealed the role of mtDYN in the pathogenesis of insulin resistance (IR) and T2D (15, 32–37). The mitochondria in T2D have been reported to be more fragmented, related to the downregulation of MFN2, in skeleton muscles from obese or T2D subjects (38–40). Also, using mice with tissue-specific knockout of Mfn2 in the liver and skeletal muscle led to fragmented mitochondrial networks, increased ROS production, and impaired metabolic features (21, 41). In T2D subjects, Mfn1 deficiency was associated with mitochondrial function impairment in the myocardium (42). In addition, venous endothelial cells isolated from T2D patients were observed with fragmented mitochondria and increased levels of Fis1 and Drp1 (43). These confirm the crucial role of mtDYN associated with obesity and T2D.

Recently, extensive studies of the mtDYN effects on immune function of both innate and adaptive immunity in antiviral response have been conducted (44, 45), but whether mtDYN could regulate innate immunity-inflammasome in response to non-infectious diseases and nutrient excess is unknown. Here, we demonstrated that mtDYN critically regulates the activity of the nutrient-sensing innate immunity-NLRP3 inflammasome pathway in diabetes-susceptible cybrid B4. Further, antidiabetic agent metformin could attenuate the pro-fission trend of mtDYN and the innate immunity-NLRP3 inflammasome pathway. We demonstrated that mitochondrial fusion proteins Mfn1 and Mfn2 are crucial for suppression of the inflammasome pathway. In the meanwhile, the fission protein Drp1 inhibitor, mdivi-1, could induce mitochondrial fusion, which also leads to reduced expression of the NLRP3 inflammasome activation.

The changes in nutrient supply and energy demand have been reported to be associated with the alterations or mutations in mtDYN. In mouse embryonic fibroblasts, nutrient excess causes mitochondrial fragmentation, while nutrient deprivation induces mitochondrial elongation (46–48). Furthermore, during nutrient shortage, mitochondria fuse to form an elongated network to maximize ATP production, whereas during nutrient excess, mitochondria divide into smaller fragments to prevent overt ATP synthesis (28). Putti et al. presented that long-chain saturated fatty acids promote skeletal muscle inflammation and IR by impairing mitochondrial bioenergetics with the fission phenotype. Conversely, omega-3 PUFAs improve insulin sensitivity in skeletal muscle by modulating mtDYN and mitochondrial function (49, 50). MtDYN may be a potential link between nutrient excess and IR (33).

Palmitate has been reported to induce ROS production in different types of cell (51–54). Increased mitochondrial fatty acid oxidation is responsible for ROS generation in lipotoxicity. The oxidation of palmitate will provide excess electrons to the mitochondrial electron transfer chain, which may give rise to overproduction of superoxide (55).

Our previous reports demonstrated impaired mitochondrial respiration and higher ROS production in the B4 cybrid in basal conditions. Besides, cybrid B4 cells have a significant lower activity of the electron transport chain complexes I, II, and V than those of cybrid D4 (4). In this study, the expressions of mitochondrial fission-related proteins and innate-immunity inflammasome were significantly increased under high fatty acid treatment in diabetes-susceptible cybrid B4 as compared to diabetes-resistant cybrid D4. It is probably that fatty acids cause increased oxidative stress and further deregulation of mitochondrial homeostasis in cybrid B4 cells, leading to a vicious circle of mitochondrial ROS. Of note, we have demonstrated a mutual interaction between mtDYN and mtROS. MtROS was reduced after overexpression of fusion-related proteins, while an opposing effect appeared after knockdown. In contrast, mitochondrial ROS increased after overexpression of Drp1 or Fis1, while an opposing effect appeared after knockdown (5). Alternatively, the fragmented mitochondrial morphology and increased mtROS in the diabetes-susceptible cybrid B4 were improved by antioxidant agent N-acetylcysteine, indicating that manipulating mtROS can alter the mtDYN (3, 4). Mitochondrial fission with a concomitant increased mtROS has been responsible to trigger NLRP3 oligomerization or to move mitochondria in close proximity to NLRP3 (56–59). The activation of the NLRP3 inflammasome then triggers the activation of downstream inflammatory cascades. In addition, palmitate was reported to induce receptor-interacting protein (RIP)1/RIP3 activation, which mediates the form of programmed lytic cell death called “necroptosis.” RIP1/RIP3 activation also leads to the activation of Drp1, resulting in ROS production and subsequent activation of NLRP3 inflammasome (60). Evidence shows that the NLRP3 inflammasome plays a pivotal role in the ischemia/reperfusion (I/R) injury, to which diabetic subjects are more sensitive (61). Ong et al. reported that the pharmacological inhibition by mdivi-1, a Drp1 inhibitor, protects the cardiac myocyte against I/R injury (62). Also, inhibition of soluble epoxide hydrolase, an enzyme responsible for the conversion of lipid epoxides to diols, significantly attenuated the NLRP3 inflammasome activation and limited the I/R injury-triggered mitochondrial localization of Drp1 (63). However, there are still some ROS-independent mechanisms. Mfn2 has been reported to be a modulator of antiviral immunity by interacting with MAVS. Overexpression of Mfn2 may lead to the inhibition of RIG-1, MDA-5, IRF-3, and NFκB (64). This explains that overexpression of Mfn2 attenuated the diet-induced inflammation in the present study. Overall, the available evidence indicates that modulation of mitochondrial dynamics could be a new therapeutic alternative for diabetic complication (62, 63, 65).

In this study, pharmacological treatment with metformin to cybrid B4 rescued the pro-fission status and consequently revealed the downregulation of the RIG-1-MAVS-NLRP3 inflammasome pathway. Metformin has been reported to attenuate the development of atherosclerosis by inhibition of mitochondrial fission mediated by Drp1 (66). Our findings are supported by the previous report, which also showed that metformin maintained mitochondrial integrity by inhibiting Drp1 activity and prevented endoplasmic reticulum (ER) stress-associated NLRP3 inflammasome activation (67). The mechanism of how metformin inhibits DRP1 may be associated with ROS modulation (68). We further tested pharmacological inhibition of fission protein Drp1 by mdivi-1, which also downregulated the RIG-1-MAVS-NLRP3 inflammasome pathway. This phenomenon has been observed in murine microglial cells in previous reports (69, 70). Our results demonstrated that both metformin and mdivi-1 ameliorate nutrient excess-induced chronic inflammation mediated through the change of mtDYN.

There are limitations in this study. First, we did not use tissues relevant for T2D in this study. However, the data showed the independent role of the mitochondrial genetic variant on innate immunity response in T2D. Metformin treatment or manipulation of mtDYN attenuated inflammation associated with nutrient excess. Second, only one siRNA was used for knockdown of Mfn-1, Mfn-2, Fis-1, and Drp-1 gene expression. Third, there is no measure of insulin action in this study. In our previous studies (4, 5), we have demonstrated the causal role of mtDYN on IR and the antioxidant agent N-acetylcysteine attenuate pro-inflammatory signal induced by insulin.

In conclusion, our results discovered the causal relationship between mtDYN and nutrient excess-induced chronic inflammation in a diabetes-susceptible cell model. Targeting mtDYN by direct interfering pro-fission can be a therapeutic intervention for chronic inflammation in T2D.

## Data Availability Statement

All datasets generated for this study are included in the article/Supplementary Material.

## Author Contributions

P-WW and S-WW developed the concept and design of study. Y-HC and S-WW wrote the manuscript with the support of H-YL and P-WW. H-YL and C-YL carried out the experiments. C-YL, J-HC, and C-WL performed the analysis. Y-HC, F-CS, Y-JS, and P-WW contributed to the interpretation of the results. H-YL and F-CS designed the figures. T-KL and Y-JS provided technical support. J-HC, T-KL, and C-WL supervised the project. All authors discussed the results and contributed to the final manuscript.

## Conflict of Interest

The authors declare that the research was conducted in the absence of any commercial or financial relationships that could be construed as a potential conflict of interest.

## Supplementary Material

Figure S1 Gene manipulation efficiency of overexpression (OE) and siRNA (si). Cybrid B4 cells transfected with indicated plasmid or small interfering dsRNA were lysed for the measurement of targeted protein level with western blot. β-actin used as loading control. Densitometric quantification normalized to corresponding vector/scramble control were presented under each blot image.

Figure S2 Perturbation of mitochondrial dynamics remodels mitochondrial activity in basal and nutrient excess. Cybrid B4 cells treated with siRNAs or mdivi-1 were exposed with the **(A)** absence or **(B)** presence of FA and ensuingly subjected to metabolic phenotyping with Seahorse XF24 bioanalyzer. **(C)** Basal, proton leak, maximal and non-mitochondrial respiration were determined by measuring OCR in the presence of basal assay medium, oligomycin (0.75 μM), FCCP (200 nM), and rotenone (0.8 μM), respectively. ECAR acquired under basal assay medium. Three independent experiments were conducted. OCR, oxygen consumption rate; ECAR, extracellular acidification rate; FA, saturated fatty acid; FCCP carbonyl cyanide-4-(trifluoromethoxy)phenylhydrazone. ^*^*p* < 0.05 compared to corresponding si-CON under identical nutrient condition. *p* < 0.05 compared to corresponding si-MFN1 under identical nutrient condition.
