# Supplementary figures and images for: The Causal Role of Mitochondrial Dynamics in Regulating Innate Immunity in Diabetes
Source: Front Endocrinol (Lausanne). 2020 Jul 29;11:445. doi: 10.3389/fendo.2020.00445 (PMC7403198; doi:10.3389/fendo.2020.00445)

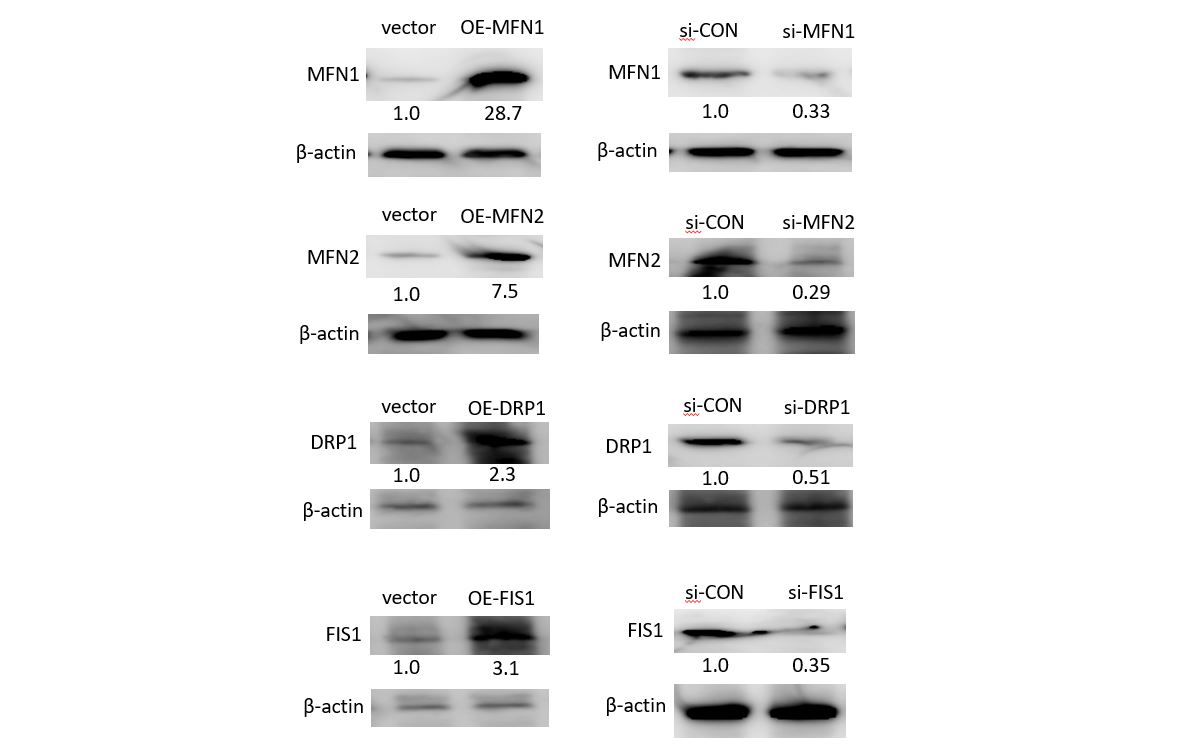

Supplement: Figure S1 — Gene manipulation efficiency of overexpression (OE) and siRNA (si). Cybrid B4 cells transfected with indicated plasmid or small interfering dsRNA were lysed for the measurement of targeted protein level with western blot. β-actin used as loading control. Densitometric quantification normalized to corresponding vector/scramble control were presented under each blot image. [file Image_1.JPEG]

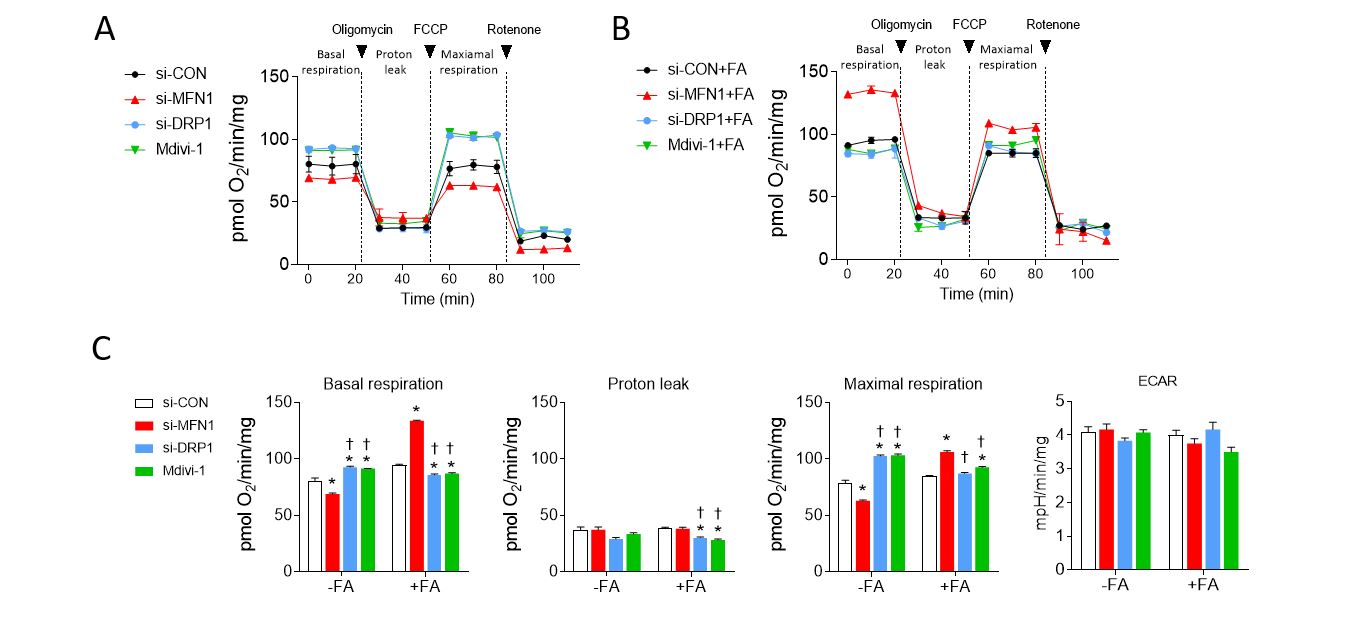

Supplement: Figure S2 — Perturbation of mitochondrial dynamics remodels mitochondrial activity in basal and nutrient excess. Cybrid B4 cells treated with siRNAs or mdivi-1 were exposed with the (A) absence or (B) presence of FA and ensuingly subjected to metabolic phenotyping with Seahorse XF24 bioanalyzer. (C) Basal, proton leak, maximal and non-mitochondrial respiration were determined by measuring OCR in the presence of basal assay medium, oligomycin (0.75 μM), FCCP (200 nM), and rotenone (0.8 μM), respectively. ECAR acquired under basal assay medium. Three independent experiments were conducted. OCR, oxygen consumption rate; ECAR, extracellular acidification rate; FA, saturated fatty acid; FCCP carbonyl cyanide-4-(trifluoromethoxy)phenylhydrazone. *p < 0.05 compared to corresponding si-CON under identical nutrient condition. p < 0.05 compared to corresponding si-MFN1 under identical nutrient condition. [file Image_2.JPEG]
